# Bone Reconstruction following Application of Bone Matrix Gelatin to Alveolar Defects: A Randomized Clinical Trial

**Published:** 2015-11-01

**Authors:** M. Bayat, F. Momen Heravi, M. Mahmoudi, N. Bahrami

**Affiliations:** 1 Oral and Maxillofacial Surgery Department, School of Dentistry, Tehran University of Medical Sciences, Tehran, Iran. Craniomaxillofacial Research Center, Tehran University of Medical Sciences, Tehran, Iran,; 2 Harvard Catalyst Laboratory for Innovative Translational Technologies, Harvard Medical School, Boston, MA, USA; 3 *Iranian Tissue Bank and Research Center, Tehran University of Medical Sciences, Tehran, Iran*

**Keywords:** Autogenous bone graft, Bone healing, Bone matrix gelatin, Bone morphogenic protein, Osteoinductive material

## Abstract

**Background:**

Conventional dentoalveolar osseous reconstruction often involves the use of graft materials with or without barrier membranes.

**Objective:**

To evaluate the efficacy of bone induction by bone matrix gelatin (BMG), delivered on an absorbable collagen sponge (ACS), compared to a placebo (ACS alone) in human alveolar socket defects.

**Methods:**

20 alveolar sockets from 10 healthy adults were studied. In all cases, both the mandibular premolar area and the contralateral premolar area (as the control site) were involved. In each of the 10 patients, the extraction sites were filled randomly with BMG and ACS. The repair response was examined on day 90. Qualitative histological and quantitative histometric analysis, including the percentage of new-formed bone fill and density were done.

**Results:**

Assessment of the alveolar bone indicated that patients treated with BMG had significantly (p<0.05) better bone quality and quantity compared to the controls. In addition, bone density and histology revealed no differences between the newly induced and native bone.

**Conclusion:**

The data from this single-blind clinical trial demonstrated that the novel combination of BMG had a striking effect on *de novo* osseous formation for the bone regeneration.

## INTRODUCTION

Dental rehabilitation of partially or totally edentulous ridges has become the primary indication for reconstruction or preservation of osseous structures to facilitate the insertion of endosseous implants. Unfavorable local conditions of the alveolar ridge following trauma, infection, developmental anomalies and surgical defects due to removal of pathologic lesions, may provide insufficient bone volume or unfavorable vertical, horizontal and sagittal intermaxillary relationships, thus rendering implant placement impossible or incorrect from a functional and esthetic viewpoint. Success rates are enhanced as increased bone volume accommodates utilizing longer and wider implants [1-3].

Autogenous bone graft has been chosen among different methods for the reconstruction, as the ideal grafting material in bone reconstructive surgery, due to its osteogenic, osteoinductive and osteoconductive properties [4-6]. Although autogenous bone graft remains the gold standard in the reconstruction of bone defects, there are disadvantages, including a limited amount of bone, donor site morbidity, hospital stay, risk of infection, and cost. The use of xenograft or alloplastic materials have therefore been developed, including silicon, polymethylmethacrylate, porous polyethylene, hydroxyapatite, deproteinized bovine bone, and tricalium phosphate. As foreign bodies, these alloplastic materials have their own inherent disadvantages including increased risk of infection and extrusion rate [7-9]. 

Restoration and regeneration of a tissue depend on proliferation and the new matrix in the injured area. Recent studies have shown successful defect reconstruction by demineralized bone matrix or bone matrix gelatin (BMG), which contains bone constructing factors such as bone morphogenic protein (BMP) [10, 11]. These proteins are soluble bone matrix glycoproteins that have the potential to act as autogenous bone graft substitutes by inducing the differentiation of osteoprogenitor cells into osteogenic cells. BMP-2, which can be produced with recombinant technology, is highly osteoinductive, inducing bone formation by stimulating the differentiation of mesenchymal cells into chondroblasts and osteoblasts [12]. Investigators have reported successful results with BMG in animals. However, to avoid bioincompatibility, human bone matrix must be evaluated. The successful use of growth factors for tissue repair is currently limited by such matters like protein stability. Using a sustained and localized delivery approach could potentially overcome this issue [13, 14]. Autolyzed antigen-extracted allogenic BMG is a matrix produced by acid demineralization of whole bone. The procedure of BMG production consists of soluble non-collagenous protein removal while native insoluble BMPs and non-collagenous proteins are kept [15-17]. 

The objective of this study was to evaluate the efficacy of bone induction by two BMGs delivered on an ACS compared to placebo (ACS alone) in human alveolar socket defect model following tooth extraction.

## MATERIALS AND METHODS

Preparation of BMG

Li, *et al* (2006), formerly described a procedure for preparing BMG. We used a similar procedure with some modifications. Briefly, bovine bone was used as bone donors for BMG preparation. All soft tissues were removed and the bones were washed in sterile deionized water. The cleaned bones were kept for 1.5 h in a 1:1 mixture of chloroform and methanol (30 mL/g of bone), and then subjected to the following steps: 1) demineralization with 0.6 M hydrochloric acid (70 mg/g) for 18–20 h; 2) washing with sterile deionized water at pH 7.4; 3) washing with 2 M CaCl_2_ for 1 h at 21 °C; 4) washing with 0.5 M ethylene-ediamine-tetra-acetic acid for 1 h; 5) washing with 8 M LiCl for 1 h; and 6) washing with deionized water at 55 °C. The BMG was then incubated with Dulbecco’s modified eagle medium supplemented with 100 U/mL penicillin, and 100 mg/mL streptomycin for 1 h at 37 °C. The resulting BMG was lyophilized and stored in a desiccator at room temperature followed by sterilization with ethylene oxide, and degassing. To get a reasonable result with BMG graft, particle sizes of 74–500 µm should be resized into 200–500 µm. Therefore, after lyophilization, BMG pieces were put in a mortar and liquid nitrogen was added to it and pieces of bone matrix were chipped by pestle. The next stage was the use of standard 250–500-µm sieves to separate suitable sized particles. Then, an accurate scale was used to weight BMG in packages of 2 mg. BMG was packed in aluminum plates and was conserved at 70 °C until planted.

Patients and Surgical Procedures

The experiment was done using 10 clinically healthy male and female patients. The patients aged between 20 and 60 years, and required contralateral tooth extraction in the mandibular premolar region and alveolar ridge preservation. They were all candidates for the present study and at the first visit, the patients were asked for their informed consent before enrollment in the study.

The inclusion criteria were systemically healthy subjects who required extraction of a premolar tooth and residual extraction sockets with less than 50% bone loss in all dimensions. The exclusion criteria were the presence of severe periodontitis or acute infections at tooth extraction, pregnancy or planning to become pregnant within one year of the experiment, recent myocardial infarction or uncontrolled bleeding disorders, the presence of mental illnesses or suspected mental illnesses, hypersensitivity to bone graft materials, and the presence of clinically significant or unstable systemic diseases affecting bone or soft tissue growth, or other renal, hepatic, endocrine, hematologic, and autoimmune diseases.

The present single-blind parallel-design randomized clinical trial was conducted at Tehran University of Medical Sciences, Tehran, Iran, from July 2011 to September 2013. The study protocol was approved by the Institutional Review Board, Tehran University of Medical Sciences.

Surgical Treatment

After administration of local anesthesia, crestal and intrasulcular incisions were made to the adjacent teeth in all patients to expose the involved teeth and alveolar crest. Extractions were performed as atraumatically as possible. The teeth were sectioned if necessary to preserve all of the socket’s bony walls. The extraction sockets were thoroughly debrided to remove all of the soft tissue. The test material was delivered through a syringe and packed into the socket by one designated oral and maxillofacial surgeon. It was passively packed after careful bleeding control with gauze. The flaps were sutured over the materials using the mattress suture technique. Primary closure was obtained using periosteal releasing incisions, if possible; minor exposure was accepted.

The medication prescribed to all subjects included antibiotics (500 mg amoxicillin 3 times daily for 3 days) and analgesics (200 mg ibuprofen 3 times daily for 5 days).

The oral wounds at the treated sites were examined at each visit, including at the baseline, days 2 and 14, and three months post-operatively, to monitor the occurrence of any of the commonly seen post-operative complications associated with the augmentation procedure (*eg*, pain, discomfort, swelling, fever, and wound dehiscence)

Post-operative Procedure

After three months, specimens from test and control sites were harvested with trephine; specimens from the defect sites were collected for histological and histometric analysis. It should be mentioned that after sampling in the extraction site, placing an implant was considered. The initial fixation of specimens in alcoholic formalin was followed by fixation in 70% ethanol for 24 h and the bone fragments were post fixed in neutral buffered formalin. The bones were then decalcified using formic acid and embedded in paraffin. Sections were prepared and stained with hematoxylin and eosin.

Measurements

One investigator blinded to the treatment received, performed the histological observations and histometric analysis using an image analysis program (NIH Image Software, Bethesda, USA). One mesial-distal central section (35-µm thick) from each defect was used for quantitative comparison of new bone formation. Histometric parameters were the percentage of bone fill and bone density in a field confined by the mesial, distal, and apical aspects of the surgically created defect and the coronal extent of induced or regenerated bone.

Statistics were presented as group means and SD. *Student’s t* test for paired samples was used for the comparison of bone density and bone filling parameters between groups. Differences between each group during treatment time points were determined by *Student’s* *t *test. A p value <0.05 was considered statistically significant.

## RESULTS

Clinical Findings

Only one case at the site of ACS developed wound failure. Antibiotics and analgesics were used by subjects during the study. Other clinical complications such as hemorrhage, abnormal swelling, and infection were not seen in any patients during the follow-up visits.

Bone Fill

The percentage of bone fill showed differences in the rates and patterns of bone formation between the two treatment groups. The percentage of bone fill at specific time points showed significantly greater levels within BMG-treated defects than ACS-treated defects (p<0.05). Scattering of data in the group of BMG-treated defects was more than that in ACS-treated defects. The mean±SD percentage of bone fill of newly formed bone after 90 days in BMG-treated defects (27.83±3.07) was significantly (p=0.044) higher than that in ACS-treated defects (22.64±2.14).

Bone Density

The percentage of bone density after 90 days in BMG-treated defects (65.45±4.54) was significantly (p=0.004) higher than that in ACS-treated defects (59.43±4.00). Data distribution was almost similar in both groups but was slightly higher in the BMG-treated defects.

Histological Observations

**Figure 1:**
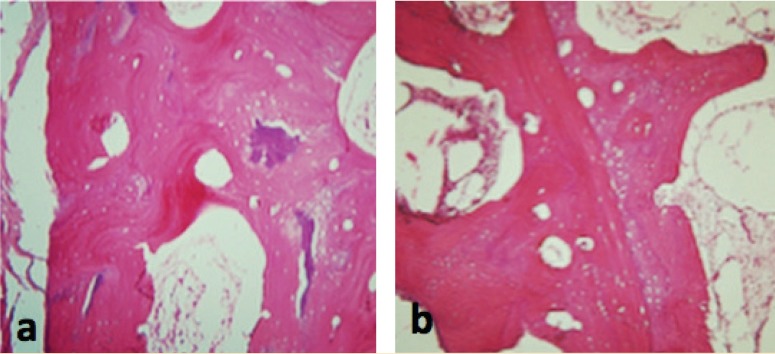
Day 90; a) bone matrix gelatin, b) absorbable collagen sponge

In the site of BMG, active remodeling of the surgical cavity was detectable in samples from the ACS sites, with normal lamellar bone structure, trabecular spaces and hematopoietic tissue. Active remodeling of the surgical cavity was detectable in samples from the BMG site, with normal cortical bone structure, bone trabeculae, and hematopoietic tissue. Newly formed bone showed recovery with normal characteristic. At the level of the medullary channel, no apparent alterations existed when the resident and healed bones were compared. From all aspects, the freshly formed bone in the BMG sites, exhibited qualities similar to that of the local bone or showed fairly dense trabeculation (Fig 1).

## DISCUSSION

Based on the studies that have shown a number of considerable advantages of BMG over other types of graft such as allogenous bone graft and other composite constructs, reconstruction of bone defects with an acid demineralized bone-derived material would be a potential treatment modality for bone formation [9].

In the present study, we treated bone defects with an acid demineralized BMG and ACS to make a comparison between them. Histological analysis revealed rather similar or better bone healing response in BMG group. Regarding bone fill measurements, BMG showed consistently greater levels at every point in time (p<0.05).

Other similar studies were performed on cats, as this study showed greater levels of bone fill at specific times within BMG-treated defects, which reached significance on days 14, 28, and 56. Regarding bone density, there was no significant difference between BMG and autogenous bone graft on days 14 and 28, but on day 56, bone density was significantly higher within the BMG-treated group. Similar to the present study, intra-treatment evaluation of bone formation in each group showed that bone density significantly increased during treatment, and bone fill in BMG group reached significance at every point in time. However, bone fill in the autogenous bone graft group only significantly increased from day 28 to 56 [14]. 

Many authors have confirmed successful defect reconstruction using demineralized bone matrix or BMG, which contains many bone constructing factors such as BMP, but one study showed that producing human BMG to avoid bioincompatibility seems to be the next step for future studies to open a new horizon in the construction of bone defects.

As a result of this study, BMG is an appropriate treatment option for the repair of bone defects that remain after surgery. The newly formed bone in the BMG implanted sites was not limited to the surface of the implanted gelatin; the new synthesized bone progressively extended in a vertical dimension along a net-like structure of BMG, which resulted in more formation of new bone. Moreover, BMG induced formation of bone trabeculae in close approximation to native bone so that it was difficult to distinguish between the implanted material and the newly formed bone.

In conclusion, though limited by a small number of subjects, the results of this study demonstrated that the BMG had striking effect on new bone formation for the insertion of dental implants. The rate of bone reconstruction using BMG is higher than that for autograft and the quality is almost similar to normal tissue. In some cases, bone resulting from BMG has a better quality so that its density is more than the natural bone.
